# Evaluation of Oxidative Stress in Dairy Cows with Left Displacement of Abomasum

**DOI:** 10.3390/ani9110966

**Published:** 2019-11-13

**Authors:** Filippo Fiore, Nicoletta Spissu, Sara Sechi, Raffaella Cocco

**Affiliations:** Department of Veterinary Medicine, University of Sassari, Via Vienna 2, 07100 Sassari, Italy; nicolettaspissu@gmail.com (N.S.); sarasechilavoro@tiscali.it (S.S.); rafco@uniss.it (R.C.)

**Keywords:** dairy cow, left displacement of the abomasum, oxidative stress, d-ROMs, BAP, OSi

## Abstract

**Simple Summary:**

The present study evaluated oxidative and antioxidant status in dairy cows with Left Displacement of the Abomasum (LDA), an economically important postpartum disease in cattle. The oxidant capacity of plasma measured with a test fo reactive oxygen metabolites, the d-ROMs test, was significantly higher and the plasma biological antioxidant potential (BAP), measured with the BAP test was lower in the LDA group compared with the control group. Oxidative status was assessed using an arbitrary index obtained from the ratio between d-ROMs and BAP and the results showed that cows with LDA experience an imbalance between oxidants and antioxidants.

**Abstract:**

Left Displacement of the Abomasum (LDA) is a condition that occurs in high-producing postpartum dairy cows and it causes economic losses. Studies performed in the last decade indicate that adult dairy cows experience oxidative stress. Increasing interest in the role of oxidative status in ruminant medicine has emphasized the need to develop reliable methods to assess it. A few studies have evaluated the relationship between LDA and oxidative status, mostly through the determination of single parameters of oxidation and the determination of antioxidant status separately, with contrasting results. The aim of this study was to assess the oxidative status by the measurement of Reactive Oxygen Metabolites with d-ROMs and Biological Antioxidant Potential BAP and the calculation of the Oxidative Status index in 74 multiparous dairy cows with LDA. Each case was matched with a control herdmate. The amount of free oxygen radicals in plasma samples was determined using the d-ROMs test, the concentration of antioxidants was measured using the BAP test and the Oxidative Status index was also calculated. The concentration of d-ROMs was significantly higher in the study group compared to the control group (179 ± 37.7 U CARR and 158 ± 23.0 U CARR, respectively), while the concentration of BAP was significantly lower in the study group than in the control group (2156 ± 98.1 µmol/L vs. 2558 ± 108.5 µmol/L). The Oxidative Status index value was significantly higher in cows with LDA than in healthy cows (8.3 ± 1.51 vs. 6.2 ± 0.76). The results of this study indicated that an inbalance between oxidants and antioxidants occurred in cattle with LDA.

## 1. Introduction

Left Displacement of the Abomasum (LDA) is a condition that occurs primarily in high-producing postpartum dairy cows [1]. LDA causes economic losses related to direct (correction, medication, discarded milk, etc.) and indirect (decreased productive and reproductive performances, increased risk of removal from the herd, etc.) costs [2]. It occurs predominantly within the first month of lactation [3], and more than 50% of cases are diagnosed in the first and second week postpartum [4,5,6].

In the postpartum period, cows experience metabolic stress as energy demands overtake energy intake, so animals undergo a state of negative energy balance (NEB) [7]. Increased lipid mobilization as a consequence of NEB may increase the generation of reactive oxygen species (ROS) [8]. Oxidative stress (OS) occurs when there is an imbalance between oxidant production and the neutralizing capacity of antioxidants, which leads to cellular damage and/or dysfunction and has been proposed as the nexus between the metabolic and immune systems of the cows during this stage [9,10]. Studies performed in the last decade indicate that adult dairy cows experience OS around the time of calving [11,12].

Increasing interest in the role of oxidative stress in ruminant medicine has emphasized the need for developing reliable methods to evaluate the systemic redox status. The methods involve the direct or indirect assessment of oxidant and antioxidant concentrations and, given the highly reactive nature of the compounds, specialized equipment is frequently required [13]. Among the commercially available kits, tests for reactive oxygen metabolites (d-ROMs) and biological antioxidant potential (BAP) have been developed to assess oxidant and antioxidant status. The d-ROMs and BAP tests, measured spetrophotometrically, have been indicated as quick, simple, precise and reliable methods for assessing oxidative status in dairy cows [14]. The identification of easy-to-use markers can be of great interest to researchers, and the possibility of assessing oxidant and antioxidant levels directly in blood using simple, reliable and cheap methods provides veterinarians with a useful tool to evaluate oxidative status in clinical situations [15].

Some authors highlighted the importance of not only evaluating concentrations of oxidants and antioxidants separately, but also analysing both components together as a ratio, to accurately detect changes in oxidant status [16]. This ratio, called Oxidative Status index (OSi), has been validated for cattle, and it is used to better assess the redox balance of the animals [17].

A few studies have evaluated the relationship between LDA and oxidative status, mostly through the determination of single parameters of oxidation and the determination of antioxidant status separately, with contrasting results [18,19,20]. Hasanpour et al. [18], Mamak et al. [21] and Aly et al. [19] found significant elevation in oxidation parameters, accompanied with a decrease in the antioxidant capacity in cows with LDA. Maden et al. [22] and Durgut et al. [23] found no significant difference in oxidants and antioxidants between healthy and LDA-affected cows. Above all, the different methods used to estimate oxidative status make it difficult to compare the results.

The objective of our study is therefore to evaluate the oxidative status by the measurement of d-ROMs and BAP with the calculation of OSi in multiparous dairy cows with LDA and in healthy control cows.

## 2. Materials and Methods

### 2.1. Animals and Management

This case-control study was carried out between January 2015 and January 2019 on cows with LDA, diagnosed by a veterinarian from the University of Sassari, in a commercial dairy farm in the north of Sardinia (Italy). The farm was composed of 280 lactating Holstein cows, housed in free-stall barns and milked twice a day. The grouping of cows into different pens was based on the stage of lactation. Dry cows were fed a “far off” total mixed ration (TMR) during the first 30–40 days of the dry period, and a “close up” TMR during the last 21 days of gestation. Lactating cows were fed twice a day a TMR that met or exceeded National Research Council requirements for 650 kg lactating cows producing 35 kg/day of milk with 3.5% fat and 3.1% protein [24] (Table 1 and Table 2).

In total, 632 Holstein dairy cows (209 primiparous and 423 multiparous cows) calved in the study period and were monitored twice a week from three weeks prepartum until diagnosis, and were on call if the farmer noticed any health problem. In addition to clinical examination, all the cows were submitted to blood testing for subclinical ketosis and subclinical hypocalcemia twice a week from calving to diagnosis. The diagnosis of LDA was based upon the presence of an acute ping sound on the auscultation and percussion of the left side of the abdomen. The inclusion criteria were: multiparous cows with an LDA independent of the degree of displacement and duration of clinical signs, less than 30 days in milk (DIM) at the time of the diagnosis, absence of concurrent diseases from three weeks prepartum to diagnosis. The concurrent diseases examined were clinical metritis, retained placenta, subclinical hypocalcemia, subclinical ketosis, clinical mastitis and lameness. Clinical metritis was defined as cows having an abnormally enlarged uterus, a fetid, watery, reddish-brown uterine discharge with fever (>39.5 °C) and the presence of signs of systemic illness (decreased milk production, dullness or other signs of toxemia) within 21 days postpartum. Retained placenta was defined as the failure to expel fetal membranes within 24 h after parturition. A blood calcium concentration of ≤2 mmol/L (8 mg/dL) was defined as subclinical hypocalcemia. Cows with Beta-Hydroxybutyrate (BHB) ≥ 1.2 mmol/L, but no clinical signs (off feed, decreased milk yield), were considered to be in a state of subclinical ketosis. Clinical mastitis was defined as the presence of abnormal milk or signs of inflammation in one or more quarters. Lameness was defined as the clinical manifestation of painful disorders, mainly related to the locomotor system, resulting in impaired movement or deviation from normal gait. It was assessed using a five-point locomotion scoring system based on both gait and posture, according to Sprecher et al. [25]. Subjects with a locomotion score ≥2 were classified as lame cows.

During the study period, 117 cows were diagnosed with LDA. Among them, 21/117 (17.9%) were excluded as they were primiparous. Of the remaining 96 multiparous cows, 22/96 (22.9%) were excluded due to concurrent diseases.

After confirmation of eligibility, 74 multiparous cows with LDA were included. Each case was matched with a control herdmate, based on age (±1 month), parity, calving date (±1 week) and 305-d milk yield (±200 kg) in the previous lactation, with an average milk yield of 9578 kg/cow/lactation. The control cows were also selected on the basis of their Body Condition Score (BCS). In particular, BCS was scored using a five-point scale, as proposed by Edmondson et al. [26], from the same trained observer for all the cows at the time of dry-off, at calving and at the time of the diagnosis. Cows were matched if they had the same BCS at dry-off and the same BCS variations from calving to diagnosis (±0.25). The mean BCS for all the animals was 3.00 ± 0.25 at dry-off, 3.25 ± 0.25 at the day of parturition and 3.00 ± 0.25 at the day of diagnosis.

The study protocol was reviewed and approved by the Ethics Committee of the University of Sassari Via Vienna 2, 07100 Sassari, Italy, with the following approval number: 28,539.

### 2.2. Blood Collection and Analyses

Blood samples were collected at the time of diagnosis from the coccygeal vein into lithium-heparinized vacutainers (10 mL) (BD, Vacutainer, Plymouth, PL6 7BP, UK). Samples were stored in a portable cooler with ice packs (approximately 4 °C) and transported to the laboratory (approximately 30 min), where they were centrifuged at 2000× *g* for 20 min. Plasma was separated and analysed immediately.

The d-ROMs and BAP concentrations were determined in a dedicated spectrophotometer (Free Radical Analytical System FRAS 4; Evolvo, Langhirano, Italy).

The amount of free oxygen radicals in plasma samples was determined using the d-ROMs test (Diacron, Grosseto, Italy), which determines hydroperoxides (the breakdown products of lipids, as well as of other organic substrates, generated by the oxidative attack of ROS) through their reaction with the chromogen *N*,*N*-diethylparaphenylenediamine. Results are expressed in arbitrary Carratelli Units (U CARR), where 1 (U CARR) is equivalent to the oxidizing power of 0.08 mg H_2_O_2_/dL.

The concentration of antioxidants was measured using the BAP test (Diacron, Grosseto, Italy). In the BAP test, the plasma samples were mixed with a colored solution obtained by mixing a ferric chloride solution with a thiocyanate derivative solution that causes a discoloration, the intensity of which is measured photometrically at 505 nm and is proportional to the ability of the plasma to reduce ferric ions. The results are expressed as µmol/L of reduced ferric ions.

The degree of oxidative stress is expressed as an Oxidative Status index (OSi) where (d-ROMs/BAP) × 100 = OSi [27].

### 2.3. Statistical Analysis

The data were analysed using R software (R Software vers. 3.3.1,The R Foundation for Statistical Computing, Vienna, Austria). All data are presented as the means ± standard error of the mean. The normal distribution of data was assessed by the D’Agostino-Pearson normality test and the analysis of statistical differences between groups was carried out using the unpaired two-tailed Student’s t-test. Differences were considered statistically significant when the *p*-value was <0.05.

## 3. Results

A total of 74 multiparous cows with LDA and 74 healthy control cows were included in our study.

The median interval between calving and the diagnosis was 11.4 ± 6.8 with a range from 1 to 29 days. Of the 74 cases, 20 were in parity 2 (27.0%), 24 were in parity 3 (32.4%) and 30 were in parity 4 (40.5%) (Table 3).

The interval from calving to LDA diagnosis was ≤7 days in 21 cows (28.4%), between 8 and 14 days in 24 cows (32.4%), between 15 and 21 days in 20 cows (27.0%) and between 22 and 30 days in nine cows (12.2%) (Table 4).

**Figure 1:**
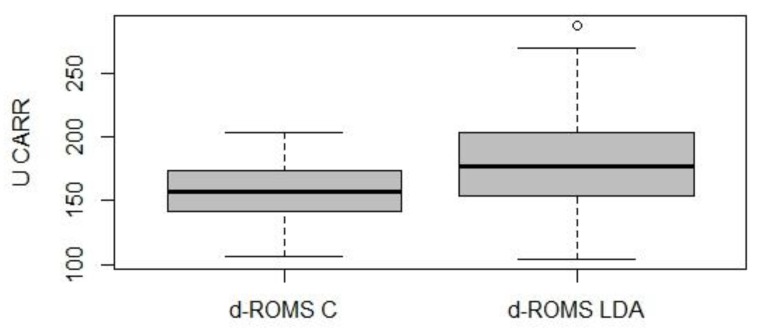
Graphical representation of the d-ROMs test’s values in the control group (*n* = 74; d-ROMS C) and in the study group (*n* = 74; d-ROMS LDA).

The concentration of d-ROMs was significantly higher in the study group compared to the control group (179 ± 37.7 U CARR and 158 ± 23.0 U CARR, respectively, *p* < 0.05) (Figure 1).

**Figure 2:**
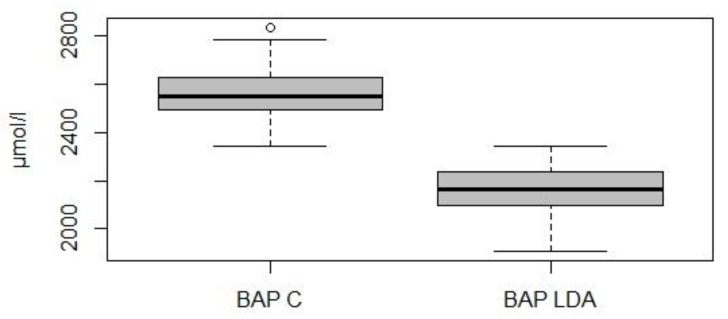
Graphical representation of the BAP test’s values in the control group (*n* = 74; BAP C) and in the study group (*n* = 74; BAP LDA).

As regards the BAP test, the concentration of antioxidants was significantly lower in the study group than in the control group (2156 ± 98.1 µmol/L vs. 2558 ± 108.5 µmol/L, *p* < 0.05) (Figure 2).

The OSi value was significantly higher (*p* < 0.05) in cows with LDA than in healthy cows (8.3 ± 1.51 vs. 6.2 ± 0.76).

## 4. Discussion

In the present study, LDA occurred most frequently in the first and second week postpartum and in parity 4 cows, which is in accordance with other studies [4,5].

The results showed a significant elevation of d-ROMs and OSi and a significant reduction of BAP in LDA cows, confirming that LDA is linked to shifts in oxidative status [18,19,20,23].

To the best of our knowledge, there is a limited number of studies on the determination of oxidative status using d-ROMs and BAP and the calculation of OSi in ruminants [28,29,30] and, in particular, these tests have never been used in cows with left abomasal displacement. We decided to calculate OSi, in addition to d-ROMs and BAP, because, in accordance with other authors, we consider that it is more important to analyse the relationship between oxidants and antioxidants through a ratio than to evaluate their concentrations separately [31]. Durgut et al. [23] published a paper regarding the changes in oxidative status associated with displaced abomasum (DA) using total antioxidant capacity and total oxidant status (TAC and TOS) to assess oxidant status and OSi as a ratio between TAC and TOS. Other researchers studied the relationship between DA and oxidative status through the determination of the serum or plasma concentrations of different oxidant (malondialdehyde, nitric oxide, etc.) and antioxidant species (superoxide dismutase, glutathione peroxidase, vitamin E, selenium, etc.) separately [18,19,21] or total antioxidant status [32,33]. Contrary to Durgut et al. [23], significantly different d-ROMs and BAP levels were found in cows with LDA and healthy controls in our study. Hasanpour et al. [18] used other methods rather than the ones used in the current work and concluded that the antioxidant status in cows with DA was lower than that of healthy controls, similarly to the findings of our study. Aly et al. [19] observed a significant elevation in the serum levels of H_2_O_2_, nitric oxide and malondialdehyde, and a significant reduction in serum catalase and glutathione reductase activities in LDA cows, confirming the induced imbalance in oxidative status linked to LDA. Unfortunately, the fact that most of the reported investigations used different analytical techniques makes it difficult to compare the data.

The results of this study indicated that an imbalance between oxidants and antioxidants occurred in cattle with left displaced abomasum. A shift in redox balance and the acute-phase reaction, as well as the inflammatory response and tissue damage, are present but moderate in LDA, because ischemia/perfusion stimulates the production of free oxygen radicals, generating compounds such as nitric oxide and malondialdehyde [22]. In addition, it was demonstrated that dairy cows with LDA suffered from peripheric fat mobilization due to energy deficits [34] and that increased lipid mobilization may increase the generation of ROS [8], so this may partly explain the significative elevation in plasma d-ROMs in the LDA group. Furthermore, a study showed that cows that developed LDA had 45% lower serum α-tocopherol concentrations than healthy cows at the last blood sampling before LDA diagnosis [20]. This is probably due to a lower Dry Matter Intake before the diagnosis. Even after vitamin E supplementation, it was possible to evidence a decrease in α-tocopherol concentration during the first week postpartum [35,36], which is thought to result from increased lipid peroxidation and the production of reactive oxygen species, increased secretion of α-tocopherol into colostrum and milk, depressed feed intake, inflammation and decreased lipid absorption and transport [37]. A lower DMI might be a probable causative factor for the lower α-tocopherol in LDA cows [20], but most probably factors other than feed intake, such as increased oxidation, play a role in lower α-tocopherol in blood around calving [38].

Given the absence of DMI data and data on the serum α-tocopherol concentrations prior to LDA in our study, we cannot determine whether lower BAP depends on α-tocopherol concentrations, lower DMI or other factors.

The mean concentrations of d-ROMs and BAP in the control group in the present study were in agreement with the findings of other studies on healthy cows in the early postpartum [15,16]. The observed changes in free radical and antioxidant concentrations appear to represent homeorhetic processes that normally occur in early lactation [29].

## 5. Conclusions

In conclusion, cows with LDA experience an imbalance between prooxidants and antioxidants when compared with healthy control cows. The plasma concentration of ROS and the plasma biological antioxidant potential were measured, using the d-ROMs test and the BAP test, respectively, in cows with LDA in the first month of lactation and in healthy control herdmates. An Oxidative Status index was also calculated. The elevation of the d-ROMs and OSi values, together with the decrease of BAP values in the diseased group, indicated that LDA is linked with a shift in oxidative status. The causes of this shift and its potential role in the pathogenesis of the disease are difficult to determine and need further research. To the authors’ knowledge, this is the first study to use d-ROMs and BAP tests, with the calculation of OSi, to investigate oxidative status in multiparous cows with LDA. This study is small, with a limited number of LDA cows in a single commercial dairy herd, so larger studies are warranted to examine the role of ROS, antioxidants and redox balance in LDA.

## Figures and Tables

**Table 1:** Ingredients of the Total Mixed Ration for lactating cows.

Ingredients	
Grass hay	34.7%
Steam flaked corn	19.0%
Cane-beet molasses blend	3.8%
Grain mix:	42.5%
- Wheat bran	29.6%
- Sorghum grain	29.4%
- Soybean meal	21.6%
- Flaked soybean	14.7%
- Calcium carbonate	2.2%
- Sodium chloride	1%
- Magnesium oxide	0.4%
- Sodium bentonite	0.9%
- Vitamin and premix:	0.3%
Vitamin A	40,000 IU
Vitamin D3	4000 IU
Vitamin E (α-tocopherol 92%)	30 mg
Vitamin B1	5 mg
Vitamin B2	3 mg
Vitamin B6	1.5 mg
Vitamin B12	0.06 mg
Vitamin K	5 mg
Para-aminobenzoic acid	5 mg
Vitamin PP (niacin)	150 mg
Choline chloride	50 mg
Fe	100 mg
Co	1 mg
I	5 mg
Mn	120 mg
Cu	10 mg
Zn	130 mg

**Table 2:** Composition of the TMR diet and the hay (mean ± Standard Deviation).

Item	TMR	Hay
DM, %	85.77 ± 0.71	88.51 ± 2.89
Ether extract, % of DM	2.48 ± 0.34	1.70 ± 0.18
Ash, % of DM	9.80 ± 0.37	8.97 ± 0.66
ADF, % of DM	20.73 ± 4.18	42.21 ± 1.71
ADL, % of DM	2.89 ± 0.63	6.74 ± 0.76
Starch, % of DM	23.60 ± 4.74	1.89 ± 2.17
CP, % of DM	14.31 ± 0.88	8.63 ± 1.17
Soluble protein, % of DM	3.89 ± 0.41	3.21 ± 0.42

DM, Dry Matter; AFD, Acid Detergent Fiber; ADL, Acid Detergent Lignin; CP, Crude Protein.

**Table 3:** Distribution of 74 cows with Left Displacement of Abomasum (LDA) as defined by lactation number.

Lactation Number	No. of Cases	Percentage of Cases (%)
2	20	27.0
3	24	32.4
4	30	40.5

**Table 4:** Time interval from calving to LDA diagnosis in 74 cows.

Time Interval (days)	No. of Cases	Percentage of Cases (%)
≤7	21	28.4
8–14	24	32.4
15–21	20	27.0
22–30	9	12.2
